# An observational study of socioeconomic disparities in psychiatry consultation uptake in Australia, using routinely collected national data from 2015 to 2022

**DOI:** 10.1177/00207640241311846

**Published:** 2025-01-18

**Authors:** Edward Meehan, Thomas Yeatman, Frances Shawyer, Darren Rajit, Vinay Lakra, Graham Meadows, Joanne Enticott

**Affiliations:** 1 Monash Centre for Health Research and Implementation, Monash University, Clayton, VIC, Australia; 2 Victorian Institute of Forensic Mental Health, Fairfield, VIC, Australia; 3 Department of Psychiatry, School of Clinical Sciences at Monash Health, Monash University, Clayton, VIC, Australia; 4 Department of Mental Health, Northern Health, Melbourne, VIC, Australia; 5 Department of Psychiatry, The University of Melbourne, Parkville, VIC, Australia; 6 Southern Synergy, School of Clinical Sciences at Monash Health, Monash University, Clayton, VIC, Australia; 7 Centre for Mental Health, School of Population and Global Health, University of Melbourne, Parkville, Victoria, Australia

**Keywords:** Psychiatry, equity, concentration index, COVID-19

## Abstract

**Background:**

The COVID-19 pandemic was associated with increased psychological distress and psychiatric service usage in Australia. Previous research into the first few months of the pandemic found severe inequality in telehealth psychiatry but no change in inequality for psychiatry service usage overall. However, it is unknown how inequality evolved over the remainder of the pandemic, as extended lockdowns continued in major Australian cities.

**Aims:**

To understand how socioeconomic inequality in psychiatric consultations changed during the COVID-19 pandemic, using new data from 2020 to 2022.

**Methods:**

We analysed routinely collected national Medicare data, provided to us as service counts per Statistical Area 3 (SA3) region by financial year from 2015 to 2016 to 2021 to 2022. We calculated daily rates of psychiatry attendances per 100,000 working age adults within each SA3 region, and we evaluated inequality in the distribution of consults using concentration indices and curves based on the Index of Relative Socio-economic Disadvantage (IRSD).

**Results:**

We analysed 7 years of Medicare data from 321 SA3 regions. The national consultation rate increased in 2020 to 2021 from 45.16 to 50.17, and then decreased slightly in 2021 to 22 to 48.65. Inequality as measured by concentration indices rose from 0.169 in 2020 to 2021 to 0.177 in 2021 to 2022. Consultation rates in the most disadvantaged IRSD quintile decreased by 15.9% in 2021 to 2022 compared to smaller decreases of between 1% and 4% in the top 4 quintiles.

**Conclusion:**

Our study shows that inequality in mental health service provision increased in the second year of the COVID-19 pandemic to the highest level seen in the 7 years of data we analysed. Individuals within the most disadvantaged IRSD quintile experienced a significant and disproportionate decline in service rates. Close monitoring and more granular data are needed to understand the drivers behind this inequity and its current status, and to inform interventions to improve it.

## Background and rationale

Since the turn of the century, successive Australian National Health Surveys have recorded a small but statistically significant increase in psychological distress, despite mental health funding doubling during the same period (Enticott et al., 2016, 2022). A disproportionate burden of psychological distress and mental disorders are known to fall on socioeconomically disadvantaged and rural populations. However, although these groups have greater treatment needs, they report lower uptake of mental health services (Enticott et al., 2016; Meadows et al., 2015). Contributing to this inequity in service uptake is the high price of co-payments (also known as ‘out-of-pockets’ or ‘gap fees’) for psychiatry consultations. Medicare, Australia’s universal healthcare insurance scheme, pays providers a scheduled fee, but an additional co-payment is often required from service users too. An analysis of Australian Bureau of Statistics (ABS) data from 2017 found that less than 30% of initial psychiatry consultations were bulk billed (i.e. did not incur a co-payment from the service user), and psychiatry had the highest yearly co-payments per person of all medical specialties, although bulk billing rates were higher for Australians in lower socioeconomic areas (Duckett et al., 2022). There are also fewer psychiatrists working in more disadvantaged rural areas compared to other areas, further compounding the barriers to access (Australian Bureau of Statistics, 2021a; Australian Government - Department of Health, 2019).

Previous research has explored the impact of the early stages of the COVID-19 pandemic on these trends, but the authors examined data only from the start of the financial year in which COVID-19 emerged (1 July 2019) to 30 November 2020 (Yeatman et al., 2023). In Australia, nationwide pandemic restrictions were first introduced in March 2020 (Hehir, 2022), but the burden of COVID-19 cases and of lockdowns subsequently diverged sharply on a state-by-state basis. Some states experienced extended lockdowns, particularly Victoria, while others, such as Tasmania, experienced only brief ‘snap-lockdowns’ (Edwards et al., 2022). The last large-scale lockdowns in Australia occurred in Victoria and New South Wales and ended in October 2021 (Edwards et al., 2022; Macreadie, 2022). During the pandemic, new Medicare Benefit Schedule (MBS) items for video-linked and phone consultations had been made available from 13 March 2020 (Savira et al., 2023). Previous research found that the video-linked MBS items were disproportionately taken up by people residing in wealthier areas (Yeatman et al., 2023). Nonetheless, overall inequality across all psychiatry attendances remained relatively stable because the unequal distribution in video-linked consultations was offset by decreasing inequality for face-to-face consultations. This was driven by pandemic restrictions which disrupted face-to-face appointments in wealthier urban areas more so than in rural areas, which were on average more disadvantaged (Yeatman et al., 2023).

However, it is not yet known how the provision and distribution of psychiatry attendances has developed since November 2020. Previous authors theorised that inequality may have worsened as COVID-19 restrictions were removed, because urban areas would continue to use more telepsychiatry and the offsetting effect of urban-centred lockdowns would evaporate (Yeatman et al., 2023).

## Aims and hypotheses

### Overarching aim

We aimed to update prior research with new data from the beginning of December 2020 until the end of June 2022 (an additional 19 months) to understand how the provision and distribution of psychiatry attendances developed during the COVID-19 pandemic in Australia. We therefore report on all Medicare funded psychiatry attendances between 1 July 2015 and 31 June 2022 (a 7-year period).

### Hypotheses

As COVID-19 led to unique changes in mental health service demand and service provision, including the introduction of video-linked sessions, we hypothesise that consultation rates and the inequality in their distribution changed significantly in 2021 and 2022, compared to the pre-pandemic era and compared to the early stages of the pandemic in 2020. We also hypothesise that inequality in the uptake of psychiatry attendances may have increased in the 2021 to 2022 financial year following the progressive relaxation of COVID-19 restrictions.

## Methods

### Study design, setting, participants, variables and data sources

We performed an observational study of routinely collected national Medicare data. The study population was comprised of patients from the financial years 2015 to 2016 up to 2021 to 2022, but data analysis was performed at the level of Statistical Area 3 (SA3) regions. Financial years in Australia run from 1 July to 30 June the following year. SA3s are geographical areas of Australia containing between 30,000 and 130,000 people, created by the ABS for the purpose of standardising regional level data comparisons. They generally align with local government and regional hubs and are clustered such that individual SA3s contain residents with similar social and economic attributes (Australian Bureau of Statistics, 2021b).

The outcome variable of interest was the rate of psychiatry attendances per 100,000 working age adults per day within each SA3 region. The exposure variables of interest were the rural-urban status and relative socioeconomic disadvantage of the SA3s.

To assess the rate of psychiatry attendances, we used aggregated data of all Medicare subsidised item codes consumed annually from Group A8 (which contains most Consultant Psychiatrist item codes) and Subgroups A4006 and A4009 (which are the item codes for Consultant Psychiatrist Telehealth and Phone attendances). The supplied data included a breakdown of consultation numbers within each SA3 region, classified based on the patient’s residential address, but no breakdowns by patient age. In calculating the consultation rate, we used ABS population estimates of working age adults in the 20 to 64 years inclusive age group.

Using the Index of Relative Socio-economic Disadvantage (IRSD) of each SA3 published by the ABS after the 2016 census, we calculated fractional ranking scores weighted by population to rank SA3s by disadvantage. The IRSD aggregates social and economic attributes of residents in each SA3, including income, occupation and education.

SA3s do not correspond exactly to Remoteness Areas published by the ABS. Therefore, to evaluate disparities between rural and urban areas, we classified all SA3s located within Greater Capital City Statistical Areas (which share the boundaries of SA3 areas) as urban and all those outside as rural. This is an imperfect approximation as some SA3s contain areas that are neither entirely rural nor entirely urban.

#### Statistical methods

Statistical analysis was performed using Stata 17.0 (StataCorp, 2021). We used the ‘merge’ function in Stata to produce a single dataset of psychiatry attendance counts, IRSD and population data and GCCSA classifications, matching based on the SA3 name and code variables. For each financial year, we calculated rates of psychiatry attendances per 100,000 working age adults per day. We reported these rates for Australia as a whole and by subclassifications for each SA3, each state and by rural-urban status. We produced choropleth maps to graphically represent the geographical distribution attendances.

We measured disparities in uptake of psychiatry attendances using concentration indices and graphically represented these with concentration curves using the ‘conindex’ function in Stata (O’Donnell et al., 2016). Concentration curves are commonly used measures of relative health inequality that can be used to plot the share of a treatment being used by cumulative proportions of a population, ranked from least advantaged to most advantaged (O’Donnell et al., 2008). The y-axis plots the health variable (from a 0% share of the use of a treatment to a 100% share) and the x-axis plots the disadvantage variable (from 0% of the population to 100%). The concentration curve will always start at the bottom left-hand corner (0, 0) and finish in the top right-hand corner (1, 1).

If a population contained no treatment disparities whatsoever between groups of different economic status, its concentration curve would be represented by a straight diagonal line, known as the ‘line of equality’. Conversely, in a population where the wealthy used more of a treatment than the poor, the concentration curve would sit below the ‘line of equality’ and vice-versa for a population where the poor used more than the wealthy. For a more visual explanation, please refer to Supplemental Figure S1, which contains an example plot that we produced to aid understanding.

To permit convenient comparisons between different concentration curves, a numeric concentration index can be calculated by doubling the area between the concentration curve and the ‘line of equality’. A positive value indicates the curve lies below the line of equality and the distribution of the health variable is concentrated among the more advantaged members of the population. A threshold of |0.2| is commonly used for indicating a high level of inequality (Araar & Duclos, 2007; Meadows et al., 2015).

#### Missing data and sensitivity analysis

When released, Medicare service counts are suppressed in SA3s with 20 or fewer consultations due to privacy concerns. The results reported in this paper were computed with the suppressed SA3s excluded entirely. We performed a sensitivity analysis to compare two other analytical approaches to the missing data: assuming that there were 20 consultations in each SA3, or assuming there were 0.

We received Medicare item code counts drawn from all ages (including patients aged under 20 years and over 64 years) and calculated consultation rates for working age Australians using working age population figures. Working age Australians use the large bulk of mental health services and are especially important to the setting of government policy. Hence, our approach was intended to provide a more reliable representation of the rate of service usage in this age group. We performed a sensitivity analysis to compare calculations using the working age population and the total Australian population, to assess how this approach influenced our findings.

## Results

### Participants and descriptive data

We requested psychiatry attendance data for all 358 SA3s, covering the whole of Australia and the external territories of Jervis Bay, Cocos Islands, Christmas Island and Norfolk Island. Item code counts were not provided in four SA3’s because of confidentiality risks due to low service counts. There was an Outside Australia SA3 and 18 non-spatial SA3 special purpose codes comprising Migratory–Offshore–Shipping and No Usual Address codes for each State and Territory. Psychiatry attendances were not provided individually for these SA3’s by the Department of Health. Instead, they were aggregated to an ‘Unknown’ observation. Both the non-spatial SA3s and the unknown observation were dropped because they could not be mapped to IRSD and working age population data. There was no available data on IRSD and working age population for 14 SA3s, so these regions were excluded from the analysis.

This left a final sample of 321 SA3’s with complete data availability, covering 7 years of Medicare data. A total of 182 SA3s (56.7%) were classified as urban and 139 (43.3%) were classified as rural. As a point of comparison, a previous paper excluded eight SA3s from analysis due to suppression of telehealth service counts (Yeatman et al., 2023). As this current paper focusses only on aggregate service counts, these eight SA3s have been re-included in the analysis, leading to slightly different estimates for consultation rates and concentration indices in 2015 through to 2020 compared to that prior paper.

### Rate of consultations per 100,000 people of working age (20–64 years) per day

As seen in Table 1, all states saw a jump in consultation rates in 2020 to 2021, coinciding with the first COVID-19 lockdowns. The most pronounced jump in consultation rates in 2020 to 2021 was in Victoria, which had the longest and strictest lockdowns of all states and territories. In 2021 to 2022, consultation rates decreased towards pre-pandemic rates in most states, but increased in Western Australia, the Northern Territory and the Australian Capital Territory.

**Table 1.** Rate of consultations per 100,000 people of working age (20–64 years) per day in Australia as a whole and by State.

Financial year	Australia	NSW	Vic	Qld	SA	WA	Tas	NT	ACT
2021 to 2022	48.65	46.34	54.76	55.81	44.49	38.12	39.49	12.57	37.09
2020 to 2021	50.17	47.82	57.17	58.46	45.67	36.24	44.82	11.13	33.99
2019 to 2020	45.16	43.22	48.80	54.39	42.91	33.13	40.73	9.60	28.18
2018 to 2019	43.97	42.52	47.92	51.81	42.48	31.55	41.57	8.93	25.57
2017 to 2018	43.45	42.04	47.97	51.03	42.79	29.23	45.06	7.27	22.47
2016 to 2017	42.98	40.67	48.14	50.71	44.10	28.46	45.39	8.05	20.13
2015 to 2016	42.34	40.69	47.53	49.12	43.86	27.52	43.89	6.96	19.26

### Consultation rates in rural and urban populations

As seen in Table 2, urban areas had consistently higher uptake of psychiatric consultations, however this disparity narrowed over time. Consultation rates increased in both populations, but while the consultation rate per person was 1.44 times as high in urban areas compared to rural areas in 2015 to 2016, it had decreased to 1.28 times as high by 2021 to 2022. Consultation rates increased by similar absolute amounts in both rural and urban areas in the first year of the pandemic, and then decreased towards (but still above) pre-pandemic baselines in 2021 to 2022, again by similar absolute amounts. A choropleth map which is illustrative of this rural-urban divide and a table of the percentage changes in rural and urban consultation rates over time are contained in the Supplemental Figure S2 and Table S1.

**Table 2.** Rate of consultations per 100,000 people of working age (20–64 years) per day in rural vs. urban locations.

Financial year	Urban	Rural
2021 to 2022	50.75	39.73
2020 to 2021	52.29	41.08
2019 to 2020	48.60	37.13
2018 to 2019	47.69	35.28
2017 to 2018	47.40	34.23
2016 to 2017	47.13	33.30
2015 to 2016	46.62	32.35

### Consultation rates by socioeconomic strata

**Figure 1.**
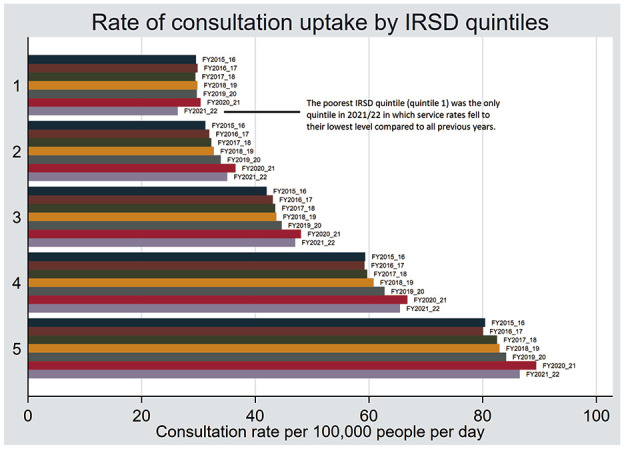
A bar graph of the rate of consultation uptake by IRSD quintiles (95% confidence intervals are small and are contained in Table 3, so they have been omitted from this graph).

**Figure 2.**
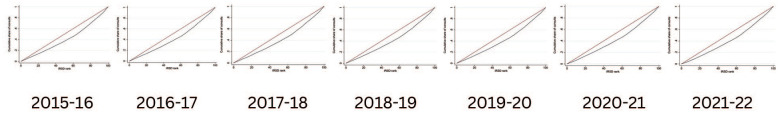
Concentration curves for nationwide uptake of psychiatry consultations by financial year. The diagonal red lines represent the line of equality. The concave blue curves underneath represent the distribution of psychiatry consultations by social disadvantage.

Consultation rates by IRSD quintile are tabulated in Table 3 and represented by bar graph in Figure 1. Figure 2 and Table 4 show the concentration curves and concentration indices respectively. The Supplemental Materials contain a table of the percentage change over time in consultation rates by IRSD quintile. Consultation rates were consistently highest in the least disadvantaged SA3s across all time periods. Notably, the most disadvantaged quintile of SA3s recorded the smallest increase in consultation rates in the first year of the pandemic (2020–2021), and the largest year on year decrease in consultation rates in the second year of the pandemic (2021–2022). The most disadvantaged quintile was also the only IRSD group in which consultation rates in 2021 to 2022 decreased to below pre-pandemic levels. This is reflected in an increased concentration index for 2021 to 2022 (see Table 4). However, the increase in the concentration index was less than one standard error.

**Table 3.** Rate of consultations per 100,000 people of working age (20–64 years) per day, 95% CI [LL, UL], by IRSD quintiles.

Financial year	Most disadvantaged	2	3	4	Least disadvantaged
2021–2022	25.14 [19.86, 30.43]	32.72 [30.44, 34.99]	48.74 [45.4, 52.07]	64.76 [59.02, 70.49]	87.63 [74.9, 100.36]
2020–2021	29.88 [23.35, 36.41]	34.13 [31.7, 36.56]	49.66 [46.13, 53.19]	65.44 [59.3, 71.57]	91.06 [77.45, 104.67]
2019–2020	29.32 [23.3, 35.34]	31.66 [29.29, 34.03]	46.43 [43.02, 49.85]	61.7 [55.71, 67.68]	86.02 [71.53, 100.5]
2018–2019	27.88 [22.58, 33.17]	30.6 [28.23, 32.96]	45.4 [42.0, 48.8]	59.6 [53.6, 65.6]	84.6 [70.65, 98.55]
2017–2018	26.91 [21.41, 32.4]	29.84 [27.36, 32.31]	45.18 [41.67, 48.69]	58.77 [52.4, 65.13]	83.79 [70.49, 97.09]
2016–2017	27.25 [21.18, 33.32]	29.8 [27.3, 32.29]	44.94 [41.32, 48.56]	57.68 [51.06, 64.29]	81.05 [67.62, 94.48]
2015–2016	26.96 [21.14, 32.78]	29.02 [26.51, 31.53]	43.66 [40.09, 47.22]	57.75 [51.09, 64.4]	80.86 [66.72, 95]

*Note.* Red text indicates a rate during the pandemic era that is lower than any of the prior yearly rates within a given socioeconomic group.

**Table 4.** Concentration indices (standard errors in brackets) for Australia as a whole and by State, by year.

Financial year	Australia	NSW	Vic	Qld	SA	WA	Tas	NT	ACT
2021–2022	0.177 (0.011)	0.196 (0.020)	0.200 (0.019)	0.167 (0.017)	0.187 (0.028)	0.174 (0.028)	0.148 (0.039)	0.153 (0.092)	0.031 (0.057)
2020–2021	0.169 (0.011)	0.191 (0.020)	0.198 (0.019)	0.149 (0.018)	0.188 (0.028)	0.180 (0.030)	0.131 (0.038)	0.090 (0.130)	−0.002 (0.057)
2019–2020	0.170 (0.012)	0.188 (0.021)	0.203 (0.019)	0.153 (0.019)	0.189 (0.029)	0.179 (0.031)	0.107 (0.046)	0.041 (0.121)	0.004 (0.059)
2018–2019	0.171 (0.012)	0.183 (0.021)	0.207 (0.019)	0.162 (0.020)	0.185 (0.029)	0.189 (0.032)	0.096 (0.048)	0.030 (0.139)	0.014 (0.059)
2017–2018	0.170 (0.012)	0.183 (0.021)	0.205 (0.019)	0.168 (0.021)	0.199 (0.031)	0.183 (0.033)	0.105 (0.048)	0.028 (0.109)	0.033 (0.066)
2016–2017	0.170 (0.013)	0.183 (0.021)	0.203 (0.019)	0.168 (0.020)	0.213 (0.034)	0.185 (0.033)	0.112 (0.055)	0.008 (0.124)	0.045 (0.071)
2015–2016	0.175 (0.013)	0.188 (0.022)	0.210 (0.021)	0.171 (0.021)	0.207 (0.034)	0.199 (0.033)	0.100 (0.051)	−0.019 (0.185)	0.046 (0.080)

### Consultation rates by state and territory

The degree of socioeconomic disparity in consultations as measured by concentration indices in different states and territories is provided in Table 4. In most years, Victoria had the highest degree of inequality. By convention, concentration indices over 0.20 are considered to represent a high level of inequality, and this threshold was surpassed in Victoria in 6 out of the 7 years of available data.

While the Australia wide concentration index increased in 2021 to 2022 compared to 2020 to 2021, there were mixed changes amongst the states. Indexes for Victoria and South Australia stayed relatively stable, while New South Wales’ index increased, and Western Australia’s decreased. The index for Queensland increased by a larger amount, as did the indexes for Tasmania, the Australian Capital Territory and the Northern Territory. In all states however, the change was less than or equal to one standard error.

### Sensitivity analyses

For our sensitivity analysis pertaining to suppressed service counts from a small number of SA3’s, results differed only slightly to the main analysis and did not alter any of our conclusions.

Our second sensitivity analysis compared calculations using the working age population and the total Australian population. The analysis showed estimated consultation rates were moderately inflated when using working age population compared to whole population figures. Use of working age population figures in our published results also produced moderately lower estimates of the concentration indices compared to estimates produced when using whole population figures in our sensitivity analysis. However, these changes applied consistently to all results, and hence trends over time and relative disparities between states and income groups were essentially unchanged.

The full results of our sensitivity analyses are contained in the Supplemental Materials.

## Discussion

### Key results and interpretations

Our objective was to evaluate how the uptake of psychiatry consultations changed in Australia as the COVID-19 pandemic entered its second year. We found that after a significant spike in the 2020 to 2021 financial year, consultation rates decreased slightly nationwide but remained above pre-pandemic levels in 2021 to 2022. In addition, we found that the nationwide distribution of psychiatry consultations became more inequitable during 2021 to 2022, mostly due to a 15.9% decrease in consultation rates in the most disadvantaged IRSD quintile, making it the only group where consultations fell below pre-pandemic levels. We also observed a significant gap in rural and urban consultations in all years, although this gap has narrowed in relative terms since 2015 to 2016.

Our results strengthen the impression that overall demand for mental health services increased due to COVID-19 and then decreased but remained above baseline levels at least into 2022. Various prior sources had also identified a rise in psychological distress and service usage in the early stages of the pandemic (Aknin et al., 2022; Australian Institute of Health and Welfare, 2021), and we found similar trends when reviewing publicly available Medicare data on psychologist attendances (Services Australia, 2024). Specifically, we retrieved MBS Group Reports for M6 and M7 (in-person psychological services) and M18 subgroups 1, 2, 6 and 7 (telehealth and phone psychological services). Total reviews rose by 1.3% from 2018–2019 to 2019–2020, then rose again by 5.6% in 2020 to 2021 (up to 6.2 million), before falling by 5.8% in 2021 to 2022, which was slightly above pre-pandemic levels. In person psychological services decreased during the pandemic, but this was compensated for by the rapid rise in telehealth and phone reviews, which grew to represent 30% of overall psychological services in 2021 to 2022, then fell to 21% of reviews in 2022 to 2023. These MBS reports do not provide a breakdown by socioeconomic or rural-urban status; hence it is not possible to assess how inequality in the uptake of psychological care, as opposed to psychiatric care, may have changed during the pandemic.

The literature suggests that drivers of increased demand for mental health services included lockdown-induced isolation and financial pressures, and fear of the virus itself (Brooks et al., 2020). The increase in consultations was also facilitated by increased supply, insofar as new MBS items for video-linked and phone consultations had been made available at the start of the pandemic (Australian Government - Department of Health, 2020; Savira et al., 2023). The decrease in consultations in 2021 to 2022 might be explained by the easing of lockdowns over the course of that year and by a populace that had become more accustomed to managing the stressors of the pandemic.

To our knowledge, this study is the first to show that the nationwide distribution of psychiatry consultations became more inequitable during the 2021 to 2022 financial year. A previous paper observed a decrease in the nationwide concentration index for the period from July to November 2020 (Yeatman et al., 2023). However, incorporating the full 12 months of data in this paper, we found that inequality remained relatively stable in 2020 to 2021 compared to previous years, before increasing in 2021 to 2022. We identified persistent inequality in the uptake of psychiatry consultations, which favoured urban and higher income Australians in all years, especially in Victoria. Placed in the context of national surveys that have reported higher psychological distress in Australians from disadvantaged backgrounds (Enticott et al., 2016), our data supports the view that Australian mental health treatment is inequitably distributed, as those with the greatest need are receiving the least consultations.

At the outset of this study, we hypothesised that inequality might increase in 2021 to 2022, as COVID-19 restrictions were progressively relaxed. This was based on predictions related to the rapid growth in video-linked telehealth appointments, which previous research showed disproportionately benefited wealthier regions (Yeatman et al., 2023). This benefit was offset in 2020 to 2021 by lockdown measures which had limited in person consultations in wealthier urban regions, but we predicted that overall inequality might rise as these lockdown measures were removed. However, we are unable to make a conclusion on this proposed mechanism because of a lack of data on the breakdown of telehealth versus in person consultations. Additionally, while we expected that video-linked consultations might increase inequality by mostly benefiting the more advantaged, it is difficult to see how this would result in the observed decrease in service use by the poorest quintile.

An alternative explanation may be that the pandemic’s financial impacts, which disproportionately burdened low wage workers (Wilkins, 2020), led Australians from the poorest quintile to reduce their consumption of psychiatry consultations. This effect was likely averted in 2020 to 2021, when COVID-19 associated government benefits and reduced household consumption led to a temporary increase in savings across all income quintiles. Between 2020 to 21 and 2021 to 2022 however, as benefits were gradually rolled back, gross savings per household fell heavily in the lowest income quintile by 61.3% to $-5,769, compared to a decrease of 2.6% across all Australians (Australian Bureau of Statistics, 2022b).

International literature suggests that the pandemic had different impacts on mental healthcare access in different settings. A global systematic review of mental health services during the pandemic found that face-to-face mental health visits generally declined while telehealth services rapidly expanded, and that a subset of patients were unreachable by telehealth due to barriers of cost, poor digital literacy and features of their mental illness (Zangani et al., 2022). Contrary to our findings, one study of emergency psychiatric presentations in Italy found a reduction in socioeconomic inequality during the pandemic (Giotta et al., 2023). Prior to the pandemic, patients from the least deprived quartile of municipalities had higher rates of psychiatric emergency department presentations than patients from poorer areas. However, during the first year of the pandemic, this rate decreased in wealthier areas while increasing in poorer areas, reducing the overall inequality in psychiatric presentations. Notably, presenting to a public hospital emergency department in Italy is free, whereas the psychiatric outpatient consultations in our study often attract a co-payment. Thus, the difference in cost barriers might explain why our findings differed to those of Giotta et al. (2023). The authors of that study also suggested that the decrease in visits in less deprived areas may have been due to greater awareness of the infectious risks of presenting to a crowded emergency department, a mechanism which is not relevant to psychiatry telehealth consults.

Despite the observed rise in psychological distress and demand for mental health treatments, suicide rates in Australia and most other countries did not increase during the pandemic (Pirkis et al., 2022). Frankl (1992) wrote that abnormal reactions to abnormal situations are normal behaviours, and for many people psychological distress might have been a natural and proportionate reaction to the profound disruption of COVID-19. Previous authors have also suggested that progression to suicide might have been averted by countervailing protective factors, including increased proximity to family members during lockdowns, increased welfare payments by governments and, importantly, better access to psychiatric treatment (Clapperton et al., 2021; Pirkis et al., 2021). If this is true, the worsening of inequality that we identified in psychiatric consultations is deeply troubling. Inequalities in protective factors against suicide could plausibly lead to inequalities in suicide itself, and a small body of research suggests that while overall suicides remained relatively stable internationally, the pandemic might have had worse impacts in lower income settings (Acharya et al., 2022; Pirkis et al., 2022). We echo previous calls for large-scale studies to assess the pandemic’s impact on suicide risk across different socioeconomic strata, and we emphasise the need to proactively consider disadvantage in the planning of suicide prevention efforts (Webb et al., 2022).

### Limitations

Our assessment of the impact on inequality of relaxing pandemic restrictions was limited by imprecision because there were still relatively lengthy lockdowns in the 2021 to 2022 financial year in several states. In the Victorian capital of Melbourne, residents spent 128 days in lockdown in the 2020 to 2021 financial year, and 91 days in lockdown in 2021 to 2022 (Macreadie, 2022). Therefore, a clean before and after comparison of the impact of lockdowns was not possible. Moreover, we did not have access to the granular Medicare data on telehealth versus in-person consultations. Consequently, we were unable to assess whether our suggested mechanism for increased inequality had occurred: that is, whether urban face to face consultations returned towards normal levels but video-linked consultations remained higher than before.

More granular data would have been ideal, including service numbers by month or by quarter, and by local government area. Item count breakdowns by age would also have been useful and would have allowed us to calculate exact working age consultation rates. However, rules governing Medicare data release stipulate that data must be suppressed if service counts are under 20 due to privacy concerns. If more granular data were used, more regions and time periods would have been suppressed.

Missing data was another limitation. Four SA3s were excluded due to the previously mentioned privacy rules, but sensitivity analysis suggested this made minimal difference to estimates. A total of 14 other SA3s were also excluded due to missing data on their IRSD and working age population. The combined population of these SA3s was very small and was unlikely to have affected the results significantly.

A possible source of confounding is the age of participants. The median age of residents is younger in Australian capital cities (which tend to be wealthier) compared to the regions (Australian Bureau of Statistics, 2022a), and both mental health and help seeking behaviours might plausibly be associated with age. However, inequality remains high when restricting to urban SA3s only. Age stratified service counts would be useful for assessing whether age is a significant confounder but would again run into issues of data suppression due to low service counts as previously outlined.

We used routinely collected Medicare data which is not produced with the requirements of medical research in mind. Item billing errors are possible, including unbilled or fraudulently billed services, or misclassification of other medical services as psychiatry consultations and vice versa, which could potentially impact on our analysis. However, there are legal penalties associated with inaccurate billing, and we consider it unlikely that misclassification significantly affected our results.

Finally, as previously discussed in our sensitivity analysis, our estimates of consultation rates among working age adults were inflated because of the inclusion in the service counts of services rendered to persons outside the 20 to 64 years age group. However, as this inflation was systemic across the entire set of results, our sensitivity analysis showed that the trends over time and relative disparities between groups remained in place when calculating total population consultation rates instead.

### Concluding remarks

Previous studies have credited increased mental health service usage as one factor among several that averted a measurable increase in suicides during the COVID-19 pandemic (Clapperton et al., 2021; Pirkis et al., 2021). However, this study is the first to establish that the most disadvantaged Australians received fewer psychiatric consultations in the latter stages of the COVID-19 era than at any point in the past 7 years, suggesting a continuing and significant social injustice. We believe that the distribution of psychiatry services, especially video-linked items, should be closely monitored and that proactive government action to address current inequities is needed.

## Supplemental Material

sj-docx-1-isp-10.1177_00207640241311846 – Supplemental material for An observational study of socioeconomic disparities in psychiatry consultation uptake in Australia, using routinely collected national data from 2015 to 2022 Supplemental material, sj-docx-1-isp-10.1177_00207640241311846 for An observational study of socioeconomic disparities in psychiatry consultation uptake in Australia, using routinely collected national data from 2015 to 2022 by Edward Meehan, Thomas Yeatman, Frances Shawyer, Darren Rajit, Vinay Lakra, Graham Meadows and Joanne Enticott in International Journal of Social Psychiatry
