# Supplementary material for: An observational study of socioeconomic disparities in psychiatry consultation uptake in Australia, using routinely collected national data from 2015 to 2022
Source: Int J Soc Psychiatry. 2025 Jan 18;71(5):945–53. doi: 10.1177/00207640241311846 (PMC12284337; doi:10.1177/00207640241311846)
Supplement: sj-docx-1-isp-10.1177_00207640241311846 – Supplemental material for An observational study of socioeconomic disparities in psychiatry consultation uptake in Australia, using routinely collected national data from 2015 to 2022 [file sj-docx-1-isp-10.1177_00207640241311846.docx]

**Supplementary Materials**

**Additional figures and tables**

*Figure S1: The following is an illustrative example of how different socioeconomic distributions of a health variable would appear on a concentration curve. The grey ‘line of equality’ represents a health variable that is shared equally amongst the whole population. The red ‘pro-rich distribution’ represents a health variable that is concentrated more amongst wealthier individuals. Finally, the blue ‘pro-poor distribution’ shows a health variable that is concentrated more amongst poorer individuals. Note that the prefix ‘pro-‘ in this context merely indicates which direction the concentration is occurring in and is not necessarily an evaluative term. The health variable within a given concentration curve may be either a positive resource (e.g., share of treatments) or a harmful health outcome (e.g., childhood mortality).*

*Figure S2: Choropleth map of the rates of consultation per 100,000 people per day in each SA3 region in 2021-22 **


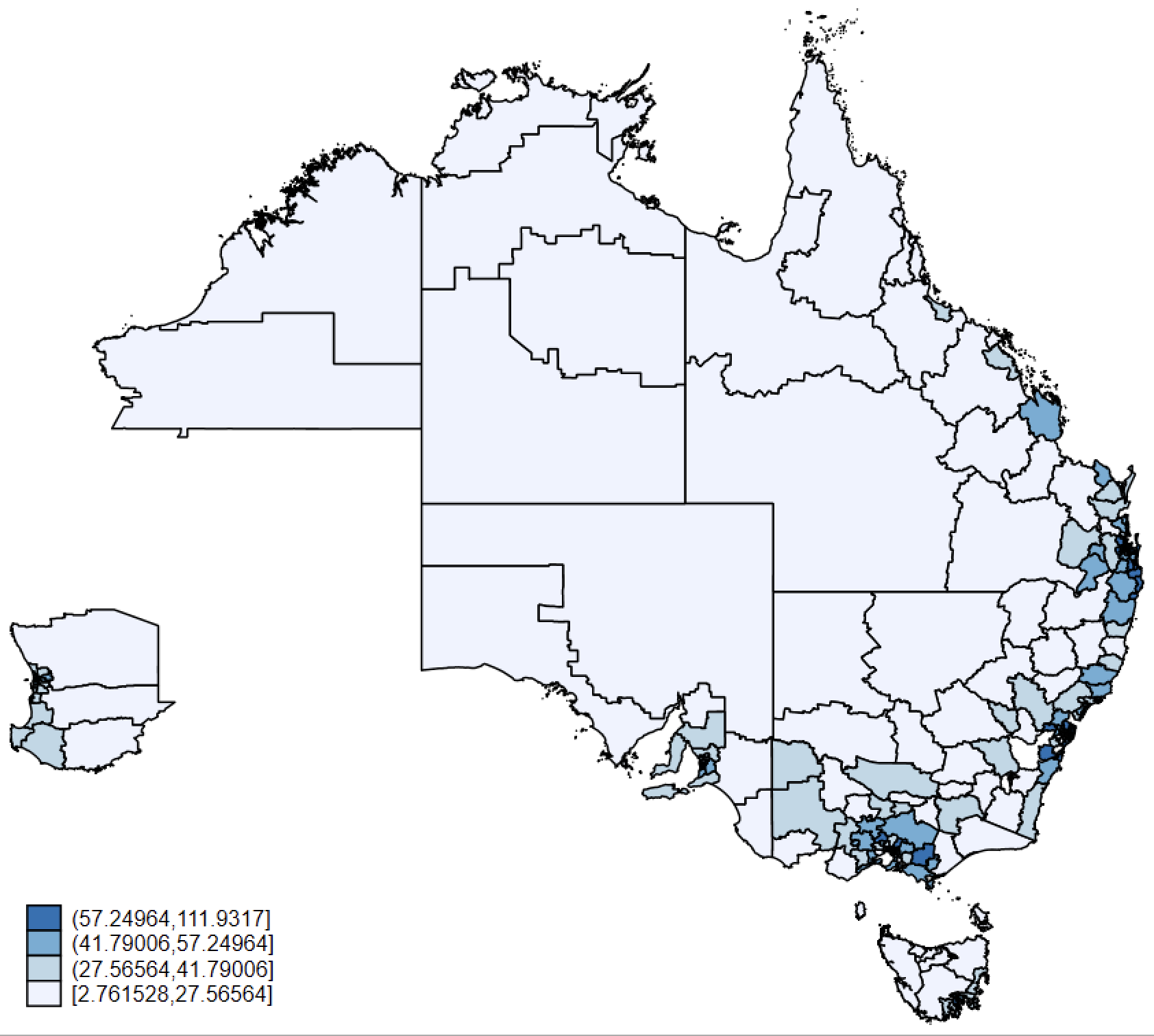

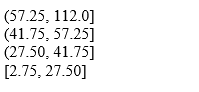


* Sparsely populated SA3’s in Western Australia were excluded due to a combination of suppressed SA3’s, missing IRSD data, and missing working age population figures.

*Table S1: Percentage change in consultation uptake over time in rural vs urban locations*

| **Comparison** | **Percentage change in consultation rate per 100,000 people of working age (20–64 years) per day** | |
| --- | --- | --- |
|  | Urban | Rural |
| From 2015-16 to 2021-22 | 8.86% | 22.81% |
| From 2019-20 to 2020-21 | 7.59% | 10.64% |
| From 2020-21 to 2021-22 | -2.95% | -3.29% |

*Table S2: Concentration indices for urban vs. rural populations, Australia wide*

| **STATE** | **Financial Year** | **Concentration Index for Total Psychiatry Consultations Australia Wide** |
| --- | --- | --- |
| Rural | 2021-22 | 0.119 (0.016) |
|  | 2020-21 | 0.103 (0.016) |
|  | 2019-20 | 0.117 (0.020) |
|  | 2018-19 | 0.122 (0.020) |
|  | 2017-18 | 0.121 (0.021) |
|  | 2016-17 | 0.121 (0.020 |
|  | 2015-16 | 0.117 (0.021) |
| Urban | 2021-22 | 0.182 (0.014) |
|  | 2020-21 | 0.176 (0.014) |
|  | 2019-20 | 0.173 (0.015) |
|  | 2018-19 | 0.169 (0.015) |
|  | 2017-18 | 0.165 (0.015) |
|  | 2016-17 | 0.162 (0.016) |
|  | 2015-16 | 0.168 (0.016) |

*Table S3: Concentration indices for urban vs. rural populations, Victoria specific*

| **STATE** | **Financial Year** | **Concentration Index for Total Psychiatry Consultations Australia Wide** |
| --- | --- | --- |
| Rural | 2021-22 | 0.107 (0.025) |
|  | 2020-21 | 0.103 (0.025) |
|  | 2019-20 | 0.084 (0.029) |
|  | 2018-19 | 0.088 (0.030) |
|  | 2017-18 | 0.076 (0.034) |
|  | 2016-17 | 0.086 (0.034) |
|  | 2015-16 | 0.091 (0.034) |
| Urban | 2021-22 | 0.210 (0.022) |
|  | 2020-21 | 0.207 (0.216) |
|  | 2019-20 | 0.213 (0.021) |
|  | 2018-19 | 0.211 (0.022) |
|  | 2017-18 | 0.205 (0.021) |
|  | 2016-17 | 0.201 (0.021) |
|  | 2015-16 | 0.208 (0.022) |

**Sensitivity Analysis for suppressed SA3’s**

*Table S4 Consultation rates by IRSD Quintile – discarding the SA3s with supressed data prior to computation*

|  | Most Disadvantaged | 2 | 3 | 4 | Least Disadvantaged |
| --- | --- | --- | --- | --- | --- |
| FY2021_22 | 25.14 (19.86-30.43) | 32.72 (30.44-34.99) | 48.74 (45.4-52.07) | 64.76 (59.02-70.49) | 87.63 (74.9-100.36) |
| FY2020_21 | 29.88 (23.35-36.41) | 34.13 (31.7-36.56) | 49.66 (46.13-53.19) | 65.44 (59.3-71.57) | 91.06 (77.45-104.67) |

*Table S5 Consultation rates by IRSD Quintile – entering 0 consultations for SA3s where the data has been suppressed.*

|  | Most Disadvantaged | 2 | 3 | 4 | Least Disadvantaged |
| --- | --- | --- | --- | --- | --- |
| FY2021_22 | 24.1 (18.6-29.59) | 32.45 (30.13-34.77) | 48.74 (45.4-52.07) | 63.68 (57.64-69.71) | 87.63 (74.9-100.36) |
| FY2020_21 | 28.64 (21.89-35.38) | 33.88 (31.42-36.34) | 49.66 (46.13-53.19) | 64.34 (57.93-70.76) | 91.06 (77.45-104.67) |

*Table S6 Consultation rates by IRSD Quintile – entering 20 consultations for SA3s where the data has been suppressed.*

|  | Most Disadvantaged | 2 | 3 | 4 | Least Disadvantaged |
| --- | --- | --- | --- | --- | --- |
| FY2021_22 | 24.93 (19.86-30) | 32.48 (30.18-34.79) | 48.74 (45.4-52.07) | 64.02 (58.2-69.85) | 87.63 (74.9-100.36) |
| FY2020_21 | 29.47 (23.18-35.76) | 33.88 (31.42-36.34) | 49.66 (46.13-53.19) | 64.69 (58.48-70.9) | 91.06 (77.45-104.67) |

*Table S7 Concentration indices for Australia – discarding the SA3s with supressed data prior to computation*

| Financial Year | Concentration Index |
| --- | --- |
| FY2021_22 | .17677769 (.01068021) |
| FY2020_21 | .16889109 (.01111806) |

*Table S8 Concentration indices for Australia – entering 0 consultations for SA3s where the data has been suppressed.*

| Financial Year | Concentration Index |
| --- | --- |
| FY2021_22 | .17683646 (.01063463) |
| FY2020_21 | .16894385 (.01106994) |

*Table S9 Concentration indices for Australia – entering 20 consultations for SA3s where the data has been suppressed.*

| Financial Year | Concentration Index |
| --- | --- |
| FY2021_22 | .17682425 (.01063285) |
| FY2020_21 | .16893861 (.01106876) |

The sensitivity analysis of the impact of different treatments of suppressed SA3s for the years 2015 to 2020 was previously reported in the supplementary materials of Yeatman 2023, and has therefore not been duplicated here.

Sensitivity analyses for state-based and rural and urban subgroups also produced results that differed minimally and had no bearing on the conclusions drawn.

**Sensitivity Analysis for working age population figures compared to total population figures**

*Table S10: Rate of consultation uptake in Australia as a whole and by State – using total population*

| **Financial Year** | **Rate of consultations per 100,000 people of working age (20–64 years) per day** | | | | | | | | |  |
| --- | --- | --- | --- | --- | --- | --- | --- | --- | --- | --- |
|  | **Australia** | NSW | Vic | Qld | SA | WA | Tas | NT | ACT |  |
| 2021-22 | 29.46 | 27.88 | 33.36 | 33.49 | 26.18 | 24.01 | 22.55 | 8.07 | 23.25 | |
| 2020-21 | 30.38 | 28.77 | 34.83 | 35.07 | 26.87 | 22.82 | 25.60 | 7.15 | 21.31 | |
| 2019-20 | 28.40 | 27.11 | 32.12 | 33.44 | 25.54 | 20.35 | 23.86 | 6.21 | 18.53 | |
| 2018-19 | 27.63 | 26.63 | 31.54 | 31.85 | 25.28 | 19.39 | 24.35 | 5.78 | 16.82 | |
| 2017-18 | 27.30 | 26.32 | 31.57 | 31.37 | 25.47 | 17.96 | 26.39 | 4.70 | 14.77 | |
| 2016-17 | 27.01 | 25.50 | 31.68 | 31.18 | 26.25 | 17.49 | 26.59 | 5.21 | 13.23 | |
| 2015-16 | 26.63 | 25.56 | 31.28 | 30.20 | 26.10 | 16.91 | 25.71 | 4.50 | 12.66 | |

*Original Table 1 from the paper: Rate of consultation uptake in Australia as a whole and by State – using working age population*

| **Financial Year** | **Rate of consultations per 100,000 people of working age (20–64 years) per day** | | | | | | | | |  |
| --- | --- | --- | --- | --- | --- | --- | --- | --- | --- | --- |
|  | **Australia** | NSW | Vic | Qld | SA | WA | Tas | NT | ACT |  |
| 2021-22 | 48.65 | 46.34 | 54.76 | 55.81 | 44.49 | 38.12 | 39.49 | 12.57 | 37.09 | |
| 2020-21 | 50.17 | 47.82 | 57.17 | 58.46 | 45.67 | 36.24 | 44.82 | 11.13 | 33.99 | |
| 2019-20 | 45.16 | 43.22 | 48.80 | 54.39 | 42.91 | 33.13 | 40.73 | 9.60 | 28.18 | |
| 2018-19 | 43.97 | 42.52 | 47.92 | 51.81 | 42.48 | 31.55 | 41.57 | 8.93 | 25.57 | |
| 2017-18 | 43.45 | 42.04 | 47.97 | 51.03 | 42.79 | 29.23 | 45.06 | 7.27 | 22.47 | |
| 2016-17 | 42.98 | 40.67 | 48.14 | 50.71 | 44.10 | 28.46 | 45.39 | 8.05 | 20.13 | |
| 2015-16 | 42.34 | 40.69 | 47.53 | 49.12 | 43.86 | 27.52 | 43.89 | 6.96 | 19.26 | |

*Table S11: Rate of consultation uptake in rural vs. urban locations – using total population*

| **Financial Year** | **Rate of consultations per 100,000 people of working age (20–64 years) per day** | |
| --- | --- | --- |
|  | **Urban** | **Rural** |
| 2021-22 | 33.17 | 22.38 |
| 2020-21 | 34.18 | 23.12 |
| 2019-20 | 32.17 | 20.95 |
| 2018-19 | 31.54 | 19.91 |
| 2017-18 | 31.34 | 19.32 |
| 2016-17 | 31.18 | 18.79 |
| 2015-16 | 30.87 | 18.25 |

*Original Table 2 from the paper: Rate of consultation uptake in rural vs. urban locations – using working age population*

| **Financial Year** | **Rate of consultations per 100,000 people of working age (20–64 years) per day** | |
| --- | --- | --- |
|  | **Urban** | **Rural** |
| 2021-22 | 50.75 | 39.73 |
| 2020-21 | 52.29 | 41.08 |
| 2019-20 | 48.60 | 37.13 |
| 2018-19 | 47.69 | 35.28 |
| 2017-18 | 47.40 | 34.23 |
| 2016-17 | 47.13 | 33.30 |
| 2015-16 | 46.62 | 32.35 |

*Table S12: Rate of consultation uptake by IRSD quintiles - using total population*

| **Financial Year** | **Rate of consultations per 100,000 people of working age (20–64 years) per day, 95% CI [LL, UL]** | | | | |
| --- | --- | --- | --- | --- | --- |
|  | Most Disadvantaged | 2 | 3 | 4 | Least disadvantaged |
| 2021-22 | 14.16, 95% CI [11.18, 17.14] | 19.12, 95% CI [17.71, 20.52] | 31.75, 95% CI [29.36, 34.14] | 42.59, 95% CI [38.39, 46.80] | 54.06, 95% CI [45.04, 63.08] |
| 2020-21 | 16.75, 95% CI [13.18, 20.33] | 19.94, 95% CI [18.44, 21.44] | 32.31, 95% CI [29.83, 34.79] | 43.05, 95% CI [38.56, 47.54] | 56.16, 95% CI [46.65, 65.68] |
| 2019-20 | 16.64, 95% CI [13.23, 20.05] | 18.57, 95% CI [17.09, 20.04] | 30.43, 95% CI [28.01, 32.86] | 40.66, 95% CI [36.32, 44.99] | 52.13, 95% CI [42.38, 61.89] |
| 2018-19 | 15.90, 95% CI [12.78, 19.03] | 17.95, 95% CI [16.48, 19.43] | 29.73, 95% CI [27.33, 32.14] | 39.27, 95% CI [34.94, 43.60] | 51.30, 95% CI [41.85, 60.74] |
| 2017-18 | 15.39, 95% CI [12.14, 18.65] | 17.51, 95% CI [15.97, 19.05] | 29.59, 95% CI [27.12, 32.06] | 38.68, 95% CI [34.17, 43.18] | 50.83, 95% CI [41.63, 60.03] |
| 2016-17 | 15.61, 95% CI [12.00, 19.22] | 17.48, 95% CI [15.92, 19.03] | 29.46, 95% CI [26.93, 31.99] | 37.91, 95% CI [33.32, 42.51] | 49.24, 95% CI [39.77, 58.71] |
| 2015-16 | 15.45, 95% CI [11.98, 18.91] | 17.02, 95% CI [15.46, 18.58] | 28.65, 95% CI [26.14, 31.15] | 37.97, 95% CI [33.32, 42.61] | 49.15, 95% CI [39.24, 59.06] |

*Original Table 3 from the paper: Rate of consultation uptake by IRSD quintiles – using working age population*

| **Financial Year** | **Rate of consultations per 100,000 people of working age (20–64 years) per day, 95% CI [LL, UL]** | | | | |
| --- | --- | --- | --- | --- | --- |
|  | Most Disadvantaged | 2 | 3 | 4 | Least disadvantaged |
| 2021-22 | 25.14 (19.86-30.43) | 32.72 (30.44-34.99) | 48.74 (45.4-52.07) | 64.76 (59.02-70.49) | 87.63 (74.9-100.36) |
| 2020-21 | 29.88 (23.35-36.41) | 34.13 (31.7-36.56) | 49.66 (46.13-53.19) | 65.44 (59.3-71.57) | 91.06 (77.45-104.67) |
| 2019-20 | 29.32 (23.3-35.34) | 31.66 (29.29-34.03) | 46.43 (43.02-49.85) | 61.7 (55.71-67.68) | 86.02 (71.53-100.5) |
| 2018-19 | 27.88 (22.58-33.17) | 30.6 (28.23-32.96) | 45.4 (42-48.8) | 59.6 (53.6-65.6) | 84.6 (70.65-98.55) |
| 2017-18 | 26.91 (21.41-32.4) | 29.84 (27.36-32.31) | 45.18 (41.67-48.69) | 58.77 (52.4-65.13) | 83.79 (70.49-97.09) |
| 2016-17 | 27.25 (21.18-33.32) | 29.8 (27.3-32.29) | 44.94 (41.32-48.56) | 57.68 (51.06-64.29) | 81.05 (67.62-94.48) |
| 2015-16 | 26.96 (21.14-32.78) | 29.02 (26.51-31.53) | 43.66 (40.09-47.22) | 57.75 (51.09-64.4) | 80.86 (66.72-95) |

*Table S13: Percentage change in consultation uptake over time by IRSD quintiles – using total population*

| **Comparison** | **Percentage change in consultation rate per 100,000 people of working age (20–64 years) per day** | | | | |
| --- | --- | --- | --- | --- | --- |
|  | Most Disadvantaged | 2 | 3 | 4 | Most advantaged |
| From 2015-16 to 2021-22 | -8.35% | 12.34% | 10.82% | 12.17% | 9.99% |
| From 2019-20 to 2020-21 | 0.66% | 7.38% | 6.18% | 5.88% | 7.73% |
| From 2020-21 to 2021-22 | -15.46% | -4.11% | -1.73% | -1.07% | 3.74% |

*Table S14a): Percentage change in consultation uptake over time by IRSD quintiles – using working age population*

| **Comparison** | **Percentage change in consultation rate per 100,000 people of working age (20–64 years) per day** | | | | |
| --- | --- | --- | --- | --- | --- |
|  | Most Disadvantaged | 2 | 3 | 4 | Most advantaged |
| From 2015-16 to 2021-22 | -6.74% | 12.76% | 11.64% | 12.15% | 8.38% |
| From 2019-20 to 2020-21 | 1.91% | 7.79% | 6.95% | 6.06% | 5.86% |
| From 2020-21 to 2021-22 | -15.86% | -4.13% | -1.85% | -1.04% | -3.77% |

*Table S14: Concentration indices for Australia as a whole and by State by year – using total population*

| **Financial Year** | **Concentration Index for Total Psychiatry Consultations** | | | | | | | | |  |
| --- | --- | --- | --- | --- | --- | --- | --- | --- | --- | --- |
|  | **Australia** | NSW | Vic | Qld | SA | WA | Tas | NT | ACT |  |
| 2021-22 | 0.197 (0.012) | 0.217 (0.021) | 0.208 (0.022) | 0.202 (0.020) | 0.187 (0.031) | 0.172 (0.028) | 0.166 (0.045) | 0.190 (0.089) | 0.070 (0.057) | |
| 2020-21 | 0.189 (0.012) | 0.212 (0.020) | 0.206 (0.021) | 0.184 (0.021) | 0.188 (0.031) | 0.179 (0.029) | 0.149 (0.045) | 0.126 (0.128) | 0.037 (0.059) | |
| 2019-20 | 0.191 (0.012) | 0.208 (0.021 | 0.211 (0.021) | 0.189 (0.022) | 0.186 (0.031) | 0.208 (0.030) | 0.128 (0.053) | 0.092 (0.116) | 0.047 (0.053) | |
| 2018-19 | 0.192 (0.013) | 0.203 (0.021) | 0.215 (0.022) | 0.198 (0.022) | 0.183 (0.032) | 0.218 (0.031) | 0.117 (0.055) | 0.081 (0.133) | 0.058 (0.052) | |
| 2017-18 | 0.192 (0.013) | 0.203 (0.022) | 0.213 (0.022) | 0.205 (0.023) | 0.196 (0.034) | 0.215 (0.032) | 0.126 (0.055) | 0.080 (0.102) | 0.077 (0.059) | |
| 2016-17 | 0.192 (0.014) | 0.203 (0.022) | 0.212 (0.022) | 0.205 (0.023) | 0.211 (0.037) | 0.216 (0.032) | 0.133 (0.063) | 0.059 (0.119) | 0.088 (0.064) | |
| 2015-16 | 0.196 (0.014) | 0.208 (0.023) | 0.220 (0.023) | 0.208 (0.024) | 0.205 (0.036) | 0.229 (0.032) | 0.120 (0.058) | 0.032 (0.179) | 0.090 (0.072) | |

*Original Table 4 from the paper: Concentration indices for Australia as a whole and by State by year – using working age population*

| **Financial Year** | **Concentration Index for Total Psychiatry Consultations** | | | | | | | | |  |
| --- | --- | --- | --- | --- | --- | --- | --- | --- | --- | --- |
|  | **Australia** | NSW | Vic | Qld | SA | WA | Tas | NT | ACT |  |
| 2021-22 | 0.177 (0.011) | 0.196 (0.020) | 0.200 (0.019) | 0.167 (0.017) | 0.187 (0.028) | 0.174 (0.028) | 0.148 (0.039) | 0.153 (0.092) | 0.031 (0.057) | |
| 2020-21 | 0.169 (0.011) | 0.191 (0.020) | 0.198 (0.019) | 0.149 (0.018) | 0.188 (0.028) | 0.180 (0.030) | 0.131 (0.038) | 0.090 (0.130) | -0.002 (0.057) | |
| 2019-20 | 0.170 (0.012) | 0.188 (0.021) | 0.203 (0.019) | 0.153 (0.019) | 0.189 (0.029) | 0.179 (0.031) | 0.107 (0.046) | 0.041 (0.121) | 0.004 (0.059) | |
| 2018-19 | 0.171 (0.012) | 0.183 (0.021) | 0.207 (0.019) | 0.162 (0.020) | 0.185 (0.029) | 0.189 (0.032) | 0.096 (0.048) | 0.030 (0.139) | 0.014 (0.059) | |
| 2017-18 | 0.170 (0.012) | 0.183 (0.021) | 0.205 (0.019) | 0.168 (0.021) | 0.199 (0.031) | 0.183 (0.033) | 0.105 (0.048) | 0.028 (0.109) | 0.033 (0.066) | |
| 2016-17 | 0.170 (0.013) | 0.183 (0.021) | 0.203 (0.019) | 0.168 (0.020) | 0.213 (0.034) | 0.185 (0.033) | 0.112 (0.055) | 0.008 (0.124) | 0.045 (0.071) | |
| 2015-16 | 0.175 (0.013) | 0.188 (0.022) | 0.210 (0.021) | 0.171 (0.021) | 0.207 (0.034) | 0.199 (0.033) | 0.100 (0.051) | -0.019 (0.185) | 0.046 (0.080) | |
